# Organellar Maps Through Proteomic Profiling – A Conceptual Guide

**DOI:** 10.1074/mcp.R120.001971

**Published:** 2020-04-28

**Authors:** Georg H. H. Borner

**Affiliations:** Department of Proteomics and Signal Transduction, Max Planck Institute of Biochemistry, 82152 Martinsried, Germany

**Keywords:** Omics, mass spectrometry, systems biology, cellular organelles, cell biology, cell fractionation, organellar proteomics, proteomics, spatial proteomics

## Abstract

Protein subcellular localization is an essential and highly regulated determinant of protein function. Major advances in mass spectrometry and imaging have allowed the development of powerful spatial proteomics approaches for determining protein localization at the whole cell scale. Here, a brief overview of current methods is presented, followed by a detailed discussion of organellar mapping through proteomic profiling. This relatively simple yet flexible approach is rapidly gaining popularity, because of its ability to capture the localizations of thousands of proteins in a single experiment. It can be used to generate high-resolution cell maps, and as a tool for monitoring protein localization dynamics. This review highlights the strengths and limitations of the approach and provides guidance to designing and interpreting profiling experiments.

## Abstract (precis)

Protein subcellular localization is highly regulated and critical for protein function. Spatial proteomics aims at capturing the localization dynamics of all proteins expressed in a given cell type. Among different approaches, organellar mapping through proteomic profiling stands out as the only method capable of determining the subcellular localizations of thousands of proteins in a single experiment. Importantly, it can also detect movements of proteins between subcellular compartments, providing an unbiased systems analysis tool for investigating physiological and pathological cellular processes.

## Graphical Abstract

**Figure:**
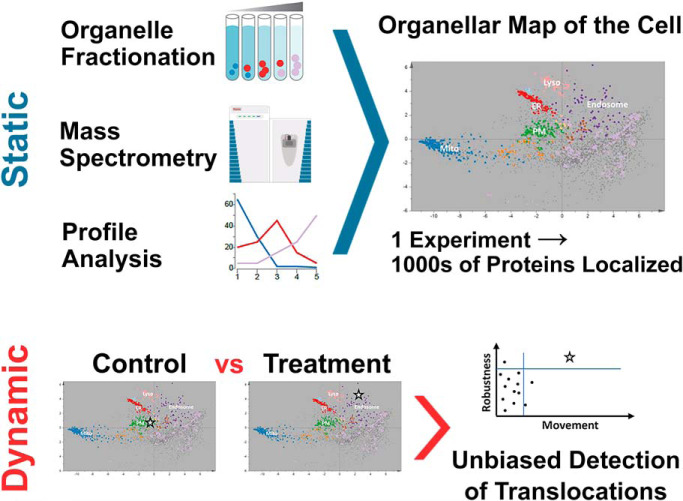

- Organelle profiling maps capture localizations of 1000s of proteins in one experiment.
- Comparing maps +/− perturbation reveals disease mechanisms & cellular responses.
- A conceptual guide to planning and interpreting organellar profiling experiments.
- A cross-study consensus set of human organellar marker proteins.

Proteins must be precisely targeted to one or more subcellular localizations, to enable them to interact with other proteins, encounter substrates, become activated or inactivated, modified, degraded, secreted, or sequestered. This spatial dimension of the proteome is carefully regulated, highly dynamic, and allows much faster responses to perturbations than alteration of gene expression. Many, perhaps most, cell biological processes involve proteins transitioning between cellular locations; evidence suggests that spatial regulation of the proteome is as important and extensive as regulation of protein abundance (1). Supporting this notion, an increasing number of diseases are associated with disturbances in protein localization (2–4). The ability to capture protein localization dynamics experimentally is hence key to understanding cellular physiology, and numerous methods for spatial proteomics are available. In this review, I will briefly summarize the state of the field, before discussing in depth the concepts, strengths, and limitations of proteomic profiling, which is arguably the simplest and fastest option for generating global organellar maps of the cell.

## 

### 

#### Current Approaches in Spatial Proteomics

There are three families of experimental approaches for spatial proteomics: imaging, interaction networks, and organellar profiling (see (5) for a detailed review). Imaging-based spatial proteomics requires a proteome-wide library of affinity reagents (6), or a comprehensive collection of cell lines expressing tagged proteins (7), as well as a set up for high-throughput microscopy. The reward is the direct visualization of each protein's localization(s) *in situ*. For spatial proteomics through interaction networks, binding partners of proteins are identified by co-immunoprecipitation (8) or the increasingly popular proximity ligation by BioID or APEX (9–11), in combination with mass spectrometry. Targeting individual compartments with multiple baits yields very comprehensive organellar inventories, which eventually connect to form a whole cell map. Again, affinity reagents or tagged cell lines are required for this approach, but not for every protein, because with enough baits the network connections begin to saturate. Organellar profiling is based on partial separation of organelles by fractionation of cell lysates (Fig. 1; (5)). The distribution of proteins across the fractions is quantified by mass spectrometry; proteins associated with the same organelle have similar abundance distribution profiles, as revealed through cluster analysis. This approach does not require any specific reagents or cell lines, is comparatively rapid and simple, and very well suited to the detection of protein translocations.

**Fig. 1.**
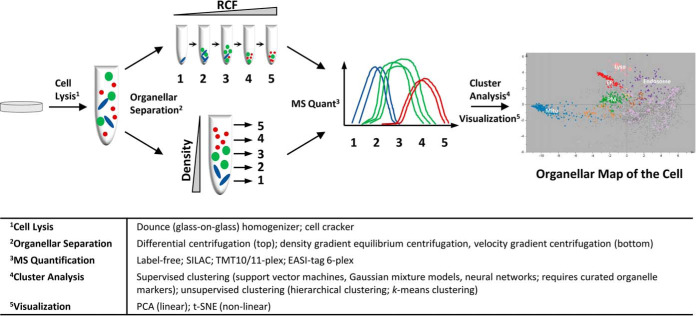
**Generic workflow for generating organellar maps through proteomic profiling.** Cells are lysed, and released organelles are partially separated by differential centrifugation (top) or density/velocity gradient centrifugation (bottom). Differential pellets or gradient fractions are analyzed by quantitative mass spectrometry. For each protein, an abundance distribution profile across the fractions is obtained. Organelles have overlapping but distinct profiles, and proteins predominantly associated with the same organelle have similar profiles. Dimensionality reduction tools (such as PCA) reveal groups of proteins with similar subcellular localization. By overlaying organellar marker proteins (color coded), the identity of clusters is revealed. Machine learning can be used to assign proteins to the nearest cluster. (Organellar map reproduced from (12)).

#### Major Databases with Subcellular Localization Information

All three of the above fields have greatly progressed in recent years, and numerous databases with subcellular localization predictions generated with different approaches are available. High throughput imaging is now relatively fast and feasible, and there is an ever-expanding repertoire of quality-controlled antibodies and GFP cell lines (6, 13). Importantly, image analysis, a previous bottleneck, is improving rapidly through automation by sophisticated machine learning algorithms (14). The Human Cell Atlas (15; https://www.proteinatlas.org/) offers an unprecedented community resource for imaging data and reagents. Proximity labeling is gradually replacing conventional affinity isolation approaches for protein interaction network analysis; the first global organellar map based on BioID data is now available (16; https://cell-map.org/) and will serve as an important framework for integrating future BioID experiments. Finally, several laboratories have applied organelle profiling to provide localization maps from diverse cell types, ranging from mammalian primary cells (17), tissues (18, 19) and cell lines (12, 20, 21) to plant cells (22) and unicellular organisms (23, 24). All these spatial proteomes are freely available, many in the form of interactive databases (Table I). In addition, several online databases collate subcellular localization information, including for example UniProt (https://www.uniprot.org), and Compartments (https://compartments.jensenlab.org/Search)).

**Table I:** Global organelle profiling studies with large subcellular localization datasets (since 2016)

Profiling method/Lab	Separation technique	Quantification strategy	Clustering method	Visualization	Cell type/tissue	Refs	Localization data available from
Protein Correlation Profiling (PCP) (Mann Lab)	Velocity gradient centrifugation	Label-free	HC, PCP, SVM	HC	Mouse liver	(18)	http://nafld-organellemap.org/
HyperLOPIT (Lilley Lab)	Density gradient centrifugation	TMT	SVM	PCA	Mouse ES cells	(20)	http://spatialmap.org/
			SVM	PCA	U2OS (human)	(15)	Supplemental Data of Ref.
			SVM	PCA, t-SNE	Arabdidopsis thaliana callus	(22)	Supplemental Data of Ref.
			SVM	PCA	Saccharomyces cerevisiae	(24)	https://proteome.shinyapps.io/yeast2018/
			SVM	PCA	Cyanobacterium synechocystis	(23)	https://lgatto.shinyapps.io/synechocystis/
LOPIT variant (Cristea Lab)	Density gradient centrifugation	TMT	RF, NN, SVM	t-SNE	Human fibroblasts	(25)	Supplemental Data of Ref.
Dynamic Organellar Maps (Borner Lab)	Differential centrifugation	SILAC	SVM	PCA	HeLa (human)	(12)	www.mapofthecell.org
		Label-free; SILAC; TMT	SVM	PCA	Mouse primary neurons	(17)	Supplemental Data of Ref.
		SILAC	SVM	PCA	MutuDC (mouse)	(26)	http://dc-biology.mrc-lmb.cam.ac.uk
LOPIT-DC (Lilley Lab)	Differential centrifugation	TMT	SVM	PCA	U2OS (human)	(27)	https://proteome.shinyapps.io/lopitdc-u2os2018/
Prolocate (Lobel Lab)	Differential and density gradient centrifugation	iTRAQ, TMT	PCP	PCA	Rat liver	(19)	http://prolocate.cabm.rutgers.edu/index.cgi
SubCellBarCode (Lehtiö Lab)	Differential centrifugation and detergent extraction	TMT	SVM	t-SNE	A431, U251, MCF7, NCIH322, HCC827 (human)	(21)	https://www.subcellbarcode.org/

Other main resources for spatial proteomes are the imaging-based Human Cell Atlas (https://www.proteinatlas.org/humanproteome/cell), the first proximity labelling based organellar map (https://cell-map.org/), the UniProt database (https://www.uniprot.org), and the Compartments database (https://compartments.jensenlab.org/Search).

Abbreviations: HC, Hierarchical Clustering; NN, Neural Networks; PCA, Principal Component Analysis; PCP, Protein Correlation Profiling; RF, Random Forest; SVM, Support Vector Machine; t-SNE, t-distributed Stochastic Neighbor Embedding; UMAP, Uniform Manifold Approximation and Projection for Dimension Reduction.

#### Choosing the Best Spatial Proteomics Approach

Different spatial proteomics approaches have pronounced strengths and drawbacks; the choice should therefore be guided by the research question, but necessarily also depends on available resources. Global approaches have intuitive appeal, but focused methods are often more appropriate, particularly if the research question only concerns a subset of the proteome, such as a single cellular compartment. Table II provides an experiment-centric overview to guide this choice; please also refer to (5).

**Table II:** Choosing the right spatial proteomics approach

Research Question			Method		Strength
Single protein	Static	Where is protein X?	Localization database (Table I)		Fast, multiple sources for cross-referencing
	Static/Dynamic	Where is protein X?	[Microscopy]		Multi-compartment localizations and transient interactions captured
		Is protein X associated with compartment Y?	Proximity labelling (APEX, BioID with protein X as bait)		
Single subcellular compartment/location	Static/Dynamic	What is the composition of compartment Y?	Proximity labelling (APEX, BioID using organelle-specific markers as baits)		Very sensitive
			Single organelle profiling		No constructs/cell lines
Global–all compartments and locations, the complete spatial proteome	Static	What is the composition of all organelles in a given cell type?	Multi organelle profiling (gradient centrifugation; long gradients for high resolution; differential centrifugation for higher throughput)		No labelling reagents, no tagging/cell line generation; relatively rapid; a single experiment covers thousands of proteins; peptide level data.
			Proximity labelling (multiple baits for every compartment)		Very sensitive, multi-compartment localizations
			Imaging (one cell line or antibody per protein)		Direct visualization, also in relation to other structures/proteins; multi-compartment localizations
	Dynamic	Which proteins change subcellular localization upon a specific perturbation, drug treatment, genetic alteration etc? Which organelles change composition upon perturbation?	*Low Resolution*	Membrane-nucleus-cytosol split	Simple, robust, deep coverage from one experiment, little MS measurement time
			*High Resolution*	Multi organelle profiling (most robust by differential centrifugation) (Proximity labelling) – no global study yet (Imaging–global studies with yeast GFP library)	Sensitive, deep coverage from one experiment

See also (5), including the supplemental data, for a detailed discussion.

Among the global mapping methods, organellar profiling is rapidly gaining popularity, for several reasons. Beyond access to mass spectrometry, it requires no special resources, and avoids the costs and potential artifacts associated with protein tagging and affinity reagents. The time commitments for a pilot study are relatively modest, and even a single experiment can reveal the localizations of thousands of proteins. Profiling also intrinsically provides protein co-fractionation data, akin to protein interaction data, allowing the detection of protein complexes (see Microclustering and Protein Complex Prediction). In addition, the approach can capture induced protein translocations, and thus provides an unbiased discovery tool to study cell biological or pharmacological processes (see Recent Applications of Comparative Organellar Profiling). Detailed protocols for mapping (28, 29) and free software for data analysis (30, 31) are also available. Organellar profiling is hence the method of choice for any lab venturing into global spatial proteomics for the first time. The remainder of this review will focus on conceptual aspects of organellar mapping by profiling, and considerations for designing and evaluating profiling experiments.

#### Evolution and Current Performance of Organellar Profiling Approaches

The origins of organellar profiling date back to the seminal work of Christian De Duve in the 1950s (reviewed in (32)). De Duve quantified enzyme activities across subcellular fractions of cell lysates obtained by ultracentrifugation, thus revealing the presence of compartments with distinct physical properties and protein compositions. In 2003, the Mann lab implemented this approach with mass spectrometry as 'Protein Correlation Profiling' (PCP), initially to characterize the composition of a single compartment, the centrosome (33). This was soon followed by the development of two related methods for profiling whole cell lysates, by the Lilley lab (LOPIT, “Localization of organelle proteins by isotope tagging”; (34, 35)), and the Mann lab (PCP; (36)). These landmark studies provided the first proteomic organellar cell maps (from Arabidopsis callus and mouse liver cells, respectively), albeit with relatively modest coverage (<1500 proteins). To date, at least six independent laboratories have developed or adapted global organellar profiling approaches (Table I). Notwithstanding individual strengths and technical differences of the methods (see Choosing the Best Design for Organellar Profiling Experiments), several general trends have emerged. Owing to improved MS instrumentation and quantification strategies, current implementations achieve coverage of >5000 proteins, in some cases with sub-compartment resolution (12, 20), and allow comparative applications (12, 18, 26, 37–39; see Recent Applications of Comparative Organellar Profiling). The number of resolved membranous compartments is around 6–10 (Table III). Good resolution is typically achieved for mitochondria, lysosomes, ER, plasma membrane, cytosol, and nucleus; depending on cell type and method, endosomes, Golgi, and peroxisomes may also be resolved. In addition, some workflows include predictions for lipid droplets, or non-organellar compartments, such as large protein complexes (*e.g.* the ribosome) and the actin cytoskeleton.

**Table III:** Compartments resolved by different implementations of organellar profiling

Refs	Profiling method	Cell type	Organelles	Other compartments
(20)	HyperLOPIT	Mouse ES cells	Endo; ER/Golgi; Lys; Mito; Nuc; Pex; PM	Cyt; ribosome; proteasome; actin cytoskeleton; extracellular matrix
(12)	Dynamic Organellar Maps	HeLa (human)	Endo; ER; ERGIC; ER_HC; Golgi; Lys; Mito; Nuc; Pex; PM	Cyt; large protein complexes; actin binding proteins
(25)	LOPIT variant	Fibroblasts (human)	ER; Golgi; Lys; Mito; Pex; PM	Cyt
(19)	Prolocate	Rat liver cells	ER; Golgi; Lys; Mito; Nuc; Pex; PM	Cyt
(18)	PCP	Mouse liver cells	Endo; ER; Golgi; Lys; Mito; Nuc; Pex; PM	Cyt; lipid droplets
(27)	LOPIT-DC	U2OS (human)	ER; Golgi; Lys; Mito; Nuc; Pex; PM	Cyt; ribosome; proteasome
(21)*	SubCellBarCode	A431, U251, MCF7, NCIH322, HCC827 (human)	Secretory 1 (Golgi, Endo/Lys); Secretory 2 (ER, Pex); Secretory 3 (ER, Mito); Secretory 4 (PM); Nuc; Mito	Cyt/cytoskeleton

Only one recent representative study is shown per laboratory and method.

Abbreviations: Endo, endosome; ER, endoplasmic reticulum; ERGIC, ER-Golgi intermediate compartment; ER_HC, ER High curvature; Golgi, Golgi apparatus; Lys, lysosome; Mito, Mitochondria; Nuc, Nucleus; Pex, peroxisome; PM, plasma membrane.

*(21) predominantly used mixed compartment classifiers.

#### Recent Applications of Comparative Organellar Profiling

Beyond providing detailed static cellular maps, a strength of organellar profiling is the ability to capture induced protein translocations (12). Comparison of maps made before and after treatment or perturbation enables the identification of proteins with altered subcellular localizations, linking them to the investigated cellular process. Several recent studies highlight the power of this approach (Table IV); examples include uncovering the molecular mechanisms of a genetic disorder (37) and obesity induced liver disease (18), capturing cellular responses during EGF signaling (12, 17, 21), the functional analysis of vesicle tethering factors (38), and the characterization of drug action (26).

**Table IV:** Applications of comparative global organellar profiling

Method	Research Question/Application	Reference
Dynamic Organellar Maps	EGF signaling	(12,17)
	Disease mechanism of AP-4 deficiency syndrome	(37)
	AP-5 mediated protein transport	(39)
	Characterization of drug action to enhance cross presentation in dendritic cells	(26)
LOPIT variant	HCMV infection	(25)
LOPIT-DC	Tethering complexes of the Golgi	(38)
PCP	Non-alcoholic fatty liver disease in mice	(18)
SubCellBarCode	EGF signaling	(21)

## Choosing the Best Design for Organellar Profiling Experiments—

Several workflows for organellar profiling experiments have been established and well documented (12, 18–21, 27). Although based on the same basic principle, they differ in key experimental steps, including cell lysis, organellar separation technique, mass spectrometric quantification approach, and data analysis. Rather than comparing individual methods directly (5), the conceptual choices open to the experimenter are discussed here. Importantly, all available methods can provide informative organellar maps, but usually offer a tradeoff: resolution and spatial information *versus* robustness and experimental simplicity. Linked to this is their suitability for static *versus* comparative applications.

### 

#### Cell Lysis

Organellar profiling requires the lysis of cells and release of ideally intact organelles. Too little lysis results in low organelle yields, whereas too much results in ruptured organelles with lumenal leakage, and potentially altered fractionation properties. Thus, lysis conditions affect both the resolution and reproducibility of a profiling experiment. Adherent culture cells (*e.g.* HeLa) tend to yield to Dounce (glass-on-glass) homogenization, which may be aided by prior gentle osmotic swelling of cells on ice (12). Alternatively, a ball-bearing homogenizer (a “cell cracker”; (20)) may be used; owing to the adjustable clearance, such a device can also cope with small suspension cells. Tissues may require tougher treatment, such as Potter-Elvehjem (teflon-on-glass) homogenization. A useful strategy during optimization is to monitor the breakage of cells with a live stain (such as trypan blue) and find the gentlest conditions that still ensure lysis of most (>90%) cells. For plant and yeast cells, enzymatic removal of cell walls prior to lysis may be helpful (22, 24). Although the addition of non-ionic detergents can render the lysis very efficient (21), it should be noted that organelles are also permeabilized. As a result, lumenal proteins become mixed with the cytosolic fraction, and organelles lose their characteristic sizes and densities.

There is intrinsic variability in the propensity of different organelles to be released from lysed cells. Small round organelles such as peroxisomes and lysosomes are more readily released than the ER, which is partly wrapped around the nucleus. This also means that the ER will always rupture partially during cell lysis. It is recommended to control organellar integrity by quantifying leakage of lumenal contents into the cytosolic fraction (12).

#### Separation of Organelles and Fractionation Schemes

Once cells have been lysed, organelles can be separated based on their physical properties, such as size and/or density. The most widely used methods include density equilibrium centrifugation (20, 29), sucrose gradient velocity centrifugation (18), differential centrifugation/pelleting (12, 21, 27, 28), or combinations thereof (19). Gradient based separation typically achieves high resolution of organelles. The downside is that it requires optimization of the gradient, relatively long centrifugation times, and substantial amounts of starting material. Furthermore, generation and harvesting of gradients is technically challenging, and introduces experimental variability, thus complicating comparative investigations (40). In contrast, differential centrifugation tends to yield somewhat lower resolution of organelles, but is technically simple, rapid, requires little starting material, and is highly reproducible.

Related to the separation method is the number of collected fractions. Typical gradient fractionations generate around 10–20 fractions, whereas differential centrifugation approaches yield only 5 to 10. More fractions tend to enhance the resolution, and long profiles may even reveal multi-organellar associations (18, 19; see Multiorganelle associations—The Bane of Profiling Based Organellar Maps). On the other hand, more samples need to be analyzed, which increases mass spectrometry measurement time, exacerbates the missing value problem, and lowers profile reproducibility. If the aim of the experiment is to establish a high resolution organellar map, a gradient based method may be best. Indeed, experimental variability between gradients of replicates may even enhance the overall resolution of the maps when data are combined (20). However, for mapping induced translocation events, robustness is more important than resolution, and differential centrifugation-based mapping thus provides a better starting point. Of note, since the introduction of the Dynamic Organellar Maps approach in 2016 (12), two other labs have independently converged on using differential centrifugation for comparative applications (21, 38), and this was indeed the favored separation technique in six out of eight recent comparative profiling studies (12, 21, 26, 37–39; Table IV).

Arguably, the simplest form of a spatial proteomics experiment is to separate cell lysates by differential centrifugation into a crude nuclear fraction, a membrane fraction, and the cytosol. Quantifying the distribution of proteins across the three fractions provides a wealth of spatial information, especially in a comparative context. Pioneered by the Lamond lab (41), several groups have now integrated this deceptively simple yet powerful workflow into their organellar profiling designs (12, 42).

#### MS Analysis and Quantification Strategy

Organellar profiling experiments are highly demanding with respect to mass spectrometry. Even for a single map, multiple fractions with quite different compositions need to be analyzed, and ideally, each identified protein should be quantified across most fractions. Furthermore, organelles tend to have overlapping profiles, which can be deconvolved only if the quantification is sufficiently accurate to resolve relatively subtle differences. The smaller the degree of organellar separation, the more accurate the quantification needs to be. For comparisons of organellar maps, profiles must be reproducible, requiring high precision of quantification. Fortunately, thanks to recent advances in instrumentation, data analysis and labeling based multiplexing strategies, there are several options to achieve this.

The simplest approach is to use label free quantification (*e.g.* using the MaxLFQ (43) algorithm; (17, 18)). This provides good sequencing depth, but the quantification is performed across multiple samples, and is intrinsically noisier than with labeling approaches. In contrast, SILAC based metabolic labeling quantification (44) offers excellent precision and accuracy for profiling, but at the cost of reduced sequencing depth per run time (17); the application of SILAC is also largely restricted to cell culture cells. Alternatively, proteins may be labeled with isobaric tags, such as TMT10/11-plex (17, 19–21, 45) and EASI-tag 6-plex (46), and thus one or even two maps may be fit into a single sample. This reduces missing values in the data but relies on consistent and efficient labeling. In addition, TMT-based quantification performed at MS2 suffers from severe ratio compression, which compromises its accuracy. Quantification at MS3 alleviates this problem (20, 47), but requires special instrumentation, and drastically slows down the sequencing speed. Thus, the advantage of multiplexing is somewhat offset by a need for extensive pre-fractionation at peptide level. Optimized acquisition modes can improve the performance of TMT (48), and a TMT 16-plex has just been released. Of note, the recently developed EASI-tag (46) allows near ratio-compression free quantification at MS2 level.

**Fig. 2.**
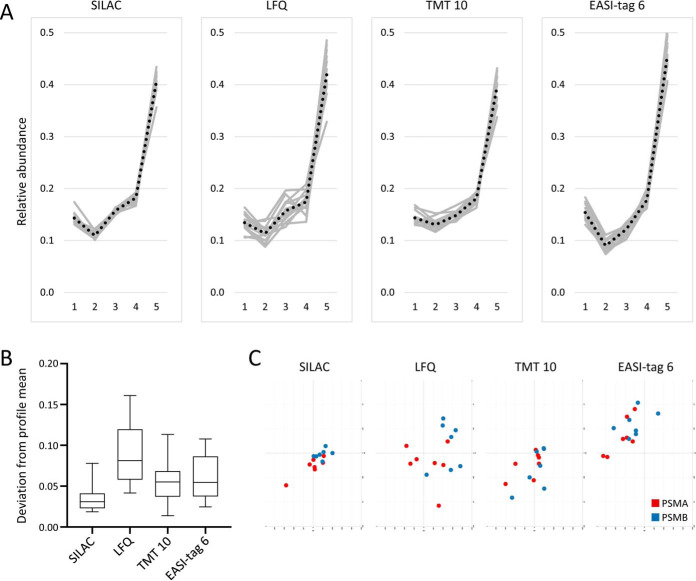
**Organellar profiling of the core proteasome performed with different MS quantification strategies.** HeLa cell lysates were fractionated into five pellets by differential centrifugation (Dynamic Organellar Maps approach (17)). The relative abundances of the 14 proteasomal core subunits (PSMA1 to PSMB7) were quantified across the fractions (normalized to sum 1). Because the core proteasome is a stable complex, the profiles are expected to be identical, and deviations largely reflect quantification error. Quantification was achieved by four different approaches (all based on data-dependent acquisition): label-free quantification using the MaxLFQ (43) algorithm; quantification against an invariant SILAC (44) heavy reference; labeling with TMT10-plex and MS3 quantification (SPS method; (47)); and labeling with EASI-tag 6-plex (46) and MS2 quantification. *A*, Relative abundance profiles; subunits in light gray, means in dashed black. SILAC quantification produces a tight profile cluster with finely nuanced resolution of small differences in the low abundance fractions (1–3). The LFQ profiles show substantially more scatter. TMT profiles are tighter than the LFQ profiles, but have a flatter shape in the first three fractions. The EASI-tag profile has the largest dynamic range. *B*, Profile scatter, *i.e.* distribution of (Manhattan) distances of the 14 profiles in A) to the average profile. Boxes show mean (line) and 1st to 3rd quartile, whiskers 5th-95th percentile; data points outside this range are not shown. SILAC quantification has the lowest scatter (smallest mean and tightest distribution), whereas LFQ has the highest scatter. TMT10 and EASI-tag 6 show similar intermediate levels. *C*, PCA plot of the 14 core proteasome subunit profiles shown in A). PCA was jointly applied to all 4 × 14 = 56 profiles, but each plot only shows the profiles obtained with the indicated quantification method; all plots have the same scale, center, and PC loadings. PSMA and PSMB subunits are color coded. SILAC quantification shows the tightest cluster, and largely resolves the A/B subcomplexes. TMT and EASI-tag show partial resolution and intermediate cluster tightness. SILAC, LFQ and TMT data were published previously (17); EASI-tag profiles were also generated in house (our unpublished data). All raw files were processed with MaxQuant (49). Importantly, the same sample set was used for LFQ, SILAC and EASI-tag quantification; for technical reasons, a very similar replicate of this set was used for TMT quantification. The LFQ profiles were obtained by reprocessing the SILAC raw files with detection of light peptides only (heavy reference channel ignored). The total MS analysis time was similar for all samples (32–40 h per map), as was the quality of instrumentation (LFQ/SILAC: Orbitrap HF; EASI-tag: Orbitrap HFX; TMT: Orbitrap Lumos).

Profiling experiments are also excellent tools to evaluate the performance of MS quantification strategies (17). Proteins that are part of a stable complex should have nearly identical profiles within the same map; any observed deviations thus largely reflect measurement noise. It is particularly informative to compare profiles from the same fractionation experiment obtained with different MS quantification methods (Fig. 2).

## Data Analysis

### 

#### Visualization of Organellar Maps

Profiling experiments typically produce high-dimensional data for thousands of proteins, which are difficult to represent in diagrammatic form. Dimensionality reduction methods compress the data down to two or three dimensions that still capture most of the information. An interpretable visual representation can be obtained by plotting the data in the reduced data space, with every protein typically displayed as a single scatter point. Proteins with similar profiles will cluster in the data map, allowing rapid discernment of resolved organelles. Software for standard visualization methods is freely available (30, 50).

There are two families of tools for dimensionality reduction - those based on linear data transformation, such as PCA (Principal component analysis), and NMF (non-negative matrix factorization; (51)); and those based on non-linear data transformation, such as t-SNE (t-distributed stochastic neighbor embedding; (52)) and UMAP (Uniform Manifold Approximation and Projection for Dimension Reduction; (53)). Linear data transformation, most notably PCA, generates similar plots for maps with similar data structure. This is important for the evaluation of replicates, or maps before/after perturbation. Also, with PCA, the position of proteins in the map can be interpreted through the loadings of the principal components. In contrast, non-linear transformation approaches (particularly the currently popular t-SNE) allow local stretching and compression of the data and tend to produce visually appealing maps with seemingly better resolved clusters. It should be noted though that the shape of the map depends strongly on the tuning parameters, can vary greatly between similar datasets, and may even produce clustering artifacts (54). This makes such maps difficult to compare across conditions and replicates.

Any visualization is a simplification that reduces the information content of the original dataset and should not be used for compartment assignment of proteins. The main purpose of a visual map is to show the overall data structure. In particular, it may help to evaluate marker proteins, gauge the shape, resolution and tightness of clusters, understand why a given protein has good or poor cluster assignment (*e.g.* positioning between two clusters), and perhaps guide the interpretation of protein translocations. Importantly, an organellar map is not an 'image' of the cell, but reflects the resolution and underlying principles of the chosen profiling approach. Organelles that appear close in the map have similar physical properties during separation, but this does not necessarily imply that these organelles are functionally similar, or physically close in the cell. Conversely, although the map shows which organelles are separated by the method, this does not preclude that these organelles are in physical contact within the cell. In fact, most organelles make contacts with other organelles (55), yet this information is largely lost during profiling.

#### Marker Proteins and Compartment Assignments

There are two main strategies for converting profiling data into lists of organellar proteins - supervised and unsupervised clustering. For the former, a list of 'bona fide' organellar marker proteins is used to outline areas of the profile space that correspond to individual organelles or subcellular structures. Next, machine learning algorithms (such as support vector machines (12, 20), Gaussian mixture models (56), neural networks and random forests (25)) are trained on the markers. The obtained models are then used to predict the association of the remaining proteins with one or more clusters. The most difficult step in this workflow is the choice of the marker set. There is currently no universally accepted panel of organellar markers; past studies have defined their own sets, usually based on “expert knowledge.” Many proteins are only expressed in certain cell types, and protein localization can also vary between cell types, so some experiment-specific curation is warranted. Further complicating matters, many if not most proteins have multiple subcellular localizations (see Multiorganelle Associations—The Bane of Profiling Based Organellar Maps). To define the profile space of an organelle optimally, proteins exclusively associated with this organelle are required. For most proteins, this information is not available a priori. Nevertheless, there are a number of proteins with fairly invariant majority steady state localization to a single organelle (*e.g.* Calnexin as an ER marker; ATP synthase as a mitochondrial marker), and an analysis of organellar metadata confirmed this notion (57). Furthermore, three independent laboratories have published organellar profiling maps from human culture cells ((12), HeLa; (25), fibroblasts; (27), U2OS), allowing the assembly of a cross-study consensus set. Predictions for seven subcellular localizations were made in all three studies (endoplasmic reticulum, Golgi, lysosomes, mitochondria, peroxisomes, plasma membrane, and cytosol). Considering only high confidence predictions (as judged by the authors), 581 proteins had consistent localization assignments across all three maps (supplemental Table S1). I propose that this set may serve as a standard core reference for future organellar mapping experiments, either as an independent test set to assess the performance of classification models, or as a useful starting point for building marker sets.

Manual curation of the marker set can be avoided using unsupervised clustering methods, allowing the profiling data first to define its own clusters, and then assigning compartments based on available external subcellular localization information of the cluster members (21). This approach is tailored to the data generated within the experiment, can be applied to cellular systems where no marker proteins have been defined, and has the potential to define novel clusters/localizations. The downside is that the definition of cluster boundaries and cluster number is arbitrary, and the resulting clusters may not correspond to actual single organelles or subcellular structures.

The observed prediction accuracy of organellar profiling, as judged by recall and precision of marker predictions, is largely determined by the choice of markers, even with cross-validation. It should be regarded as an estimate of the confidence of predictions purely within the cluster boundaries defined by the given marker set, but not as an absolute measure of organellar resolution. Comparing prediction performances across studies is hence of limited value (unless estimates are based on the same independent validation set of marker proteins, which must not have been used for training). However, F1 scores (the harmonic mean of recall and precision) and the corresponding “confusion matrix” of wrongly assigned marker proteins are very useful experiment-internal metrics, particularly for map optimization and interpretation, as they highlight which organellar mis-assignments are likely to occur.

#### Multiorganelle Associations—The Bane of Profiling Based Organellar Maps

Many, if not most, proteins have multiple subcellular localizations, ranging from transient movements during biosynthesis, to functional re-localizations, and genuine multi-compartment functions or even moonlighting activities (5). Examples include proteins shuttling between nucleus and cytosol, proteins with both membrane-bound and cytosolic pools, and proteins cycling within the endomembrane system. However, profiling experiments capture only a majority steady state snapshot of protein abundance distribution. The profile of a protein that predominantly associates with a single compartment will closely correspond to the theoretical compartment profile. Thus, its association will be predicted with high confidence. If the protein has a second or third minor location, the profile will be peripheral to that of the main cluster. In case of a genuine bimodal distribution, the profile will lie between the two organellar clusters, resulting in a poor prediction confidence for the protein. The intermediate location is determined by the “geography” of the map, *i.e.* the relative positions of the two organelles. In the worst case, a third organelle lies in between the two clusters, and the protein is miss-assigned.

For these reasons, profiling maps tend to generate a relatively large proportion of profiles that are not confidently assigned to a single compartment (typically 30–50%). The very fact that a protein is poorly assigned has been interpreted as evidence of multiple localizations (20, 21). Some organellar profiling approaches include features to alleviate the multi-localization problem. The Dynamic Organellar Maps approach for example quantifies the cytosolic *versus* organellar pools of proteins independently from their compartment assignments, allowing dual localization predictions for proteins that are partly organellar and partly cytosolic (12). Furthermore, profiles obtained from long gradients with a large number of subfractions (*e.g.* (18); 22 fractions) can to some extent resolve bimodal protein distributions, indicating two major steady state localizations. Multimodal distributions are generally more difficult to observe with short gradients and few fractions (<10). Although it is computationally possible to deconvolve an observed profile into multiple underlying organellar profiles with different weights (19), it should be noted that this is difficult to control. Firstly, the “pure” profiles of the different compartments are not known, and must be estimated from marker protein averages. Secondly, fitting two or more organelle profiles to account for a presumed mixed profile is almost always superior to fitting a single organelle profile, but the danger of overfitting is high. Most importantly, there are almost no orthogonal quantitative data available to calibrate or benchmark such deconvolution attempts. If quantification of a sufficient number of marker proteins by imaging was combined with a proteomic profiling study of the same cells, accurate deconvolution of the underlying “pure” compartment profiles may be possible; however, this has not been reported to date.

The limited prediction of multiorganelle association is a downside of profiling approaches. Nevertheless, any profile, interpretable or not, provides spatial information. This is particularly relevant for comparative experiments (see below, Detection of Protein Translocations); because profile shifts are evaluated, all profiled proteins can be included, even those lacking a confident cluster assignment.

#### Microclustering and Protein Complex Prediction

Proteins that are part of a stable complex have tightly linked fractionation profiles, resulting in visible “microcluster” formation within organellar maps (12, 20, 21). Conversely, this can be exploited to predict new protein complexes (12, 21; see (58) for a detailed discussion). This works only “one-way” though. If proteins are clearly co-fractionating, it suggests that they have a linked distribution in the cell, as typical of proteins within a complex. If two proteins have dissimilar profiles, it shows that they are not predominantly co-distributed, but does not rule out a transient or partial interaction. Profiling is hence a good way to identify stable protein complexes (58). The ability of profiling to do so is governed by the accuracy and precision of the quantification method (see MS Analysis and Quantification Strategy, and Fig. 2*C*), and greatly improves with multiple replicates.

#### Detection of Protein Translocations

A main application of organellar mapping is to identify proteins with shifted subcellular localization following a perturbation. An intuitive approach is to focus on proteins with altered predicted compartment association, but this is rarely advisable. First, it requires that both compartment association predictions are correct, and hence may produce false positives. Second, it can only detect near complete organellar translocations; a partial (or even intra-organellar) shift is unlikely to alter the original compartment association prediction, resulting in false negatives. Evidence suggests that many translocations are partial (see for example (38)) and would therefore escape detection. A much more sensitive approach is to evaluate profile shifts, regardless of compartment assignment. The profile is also quantitative, rather than qualitative, and hence amenable to multivariate statistical analysis. A straightforward approach is to subtract the profile before and after perturbation for each protein (12). For proteins not changing localization, these “delta” profiles should reflect experimental noise. Significant outliers from this distribution are likely candidate translocating proteins (12, 37, 38, 39). Further quality filters may be included, such as evaluating reproducibility of the shift direction (12, 37, 38, 39). The nature of the shift (from where to where) may subsequently be interpreted considering compartment association predictions or visual maps. Even in cases where this is not conclusive, the detection of the translocation event itself is arguably more important, as it indicates relevant proteins for orthogonal validation (see Interpretation of Profile Shifts, Orthogonal Validation).

Of note, seven out of eight published comparative profiling studies have analyzed profile shifts to detect translocations (12, 18, 21, 26, 37–39; Table IV), so this is currently the consensus approach. Only in cases where the perturbation causes extreme morphological changes (and hence strongly altered profiles of most organelles), it may be better to rely on altered compartment association predictions (as in (25)), albeit at the price of lower sensitivity and specificity.

Because comparative profiling experiments are intrinsically noisy, FDR control for the detection of translocations is essential. A good way to calibrate the experimental system is to perform a 'mock' comparative experiment, *i.e.* to compare two sets of (untreated) replicate maps. At a given set of cut-offs, the number of hits in the mock experiment is divided by the number of hits in the perturbation experiment, yielding an FDR estimate (12, 25). At least two replicates of control and perturbation maps are typically required for useful FDR levels. With three or more replicates, FDRs close to 0 have been achieved (12, 37).

Although it would be highly desirable to estimate the sensitivity of comparative profiling experiments, this has not been possible to date. Because such experiments typically aim to uncover novel connections, the 'ground truth' is not known a priori. If possible, positive controls that are expected to shift strongly upon perturbation should be part of the experimental design. These help to gauge the sensitivity and to adjust stringency cut-offs.

#### Interpretation of Profile Shifts, Orthogonal Validation

Profiling translocation analysis is a reductionist tool - its greatest strength is to identify from thousands of profiled proteins a relatively small set specifically responding to a perturbation. The observed shifts may be directly interpretable regarding spatial rearrangements, both by comparing organellar assignments, shifts in visual maps, and across cytosolic, membrane and nuclear fractions (12). However, partial movements are often not directly interpretable. The elegant application of organellar mapping to identify vesicle proteins rerouted onto Golgi tethers anchored to mitochondria is a case in point (38). Although substantial numbers of vesicles were captured on mitochondria, as demonstrated by microscopy, the organellar maps showed only moderate shifts of vesicle proteins. Without additional information, these would not have been conclusive; but in the context of the experimental design and orthogonal data, they revealed a highly specific set of vesicle proteins cognate to the tested Golgi tethers. Similarly, Davies *et al.* (37) identified ATG9A as a cargo protein of AP-4 vesicles, through profiling of AP4 knockout cells. The detected shifts were suggestive of TGN retention of ATG9A, but only microscopy of AP-4 knockout and patient derived cells demonstrated unambiguously that this is the case. These studies illustrate the power of comparative profiling to find the proverbial needle in a haystack, but also highlight that the most informative approach is to combine global profiling with further targeted characterization of the detected changes, especially through imaging.

## Perspective—

Comparative organellar profiling promises to become a widespread systems analysis tool, with countless potential applications in cell biology, pharmacology and biomedical research. Although high quality MS instrumentation is now available to many labs, the considerable MS time requirements for profiling are still the biggest bottleneck; consequently, most profiling based publications are restricted to binary comparisons. A priority in the field should be the development of streamlined workflows that allow fast pilot experiments, more high-throughput mapping, and more complex experimental designs.

The rapidly increasing number of spatial proteomics studies is exciting and shows the growing momentum of the field. A potential concern is that the current databases of protein subcellular localization cannot be easily cross-referenced. There is no central repository, and no universal data format that allows rapid comparisons of the predictions. Database curators, or perhaps the proteomic community, should tackle these issues soon, to maximize accessibility of current and future spatial proteomics datasets.

## Supplementary Material

Table S1
